# Carbon Sink Potential of Sulfur-Oxidizing Bacteria in Groundwater at Petroleum-Contaminated Sites

**DOI:** 10.3390/microorganisms13071688

**Published:** 2025-07-18

**Authors:** Pingping Cai, Zhuo Ning, Min Zhang

**Affiliations:** 1 School of Water Resources and Environment, Hebei GEO University, Shijiazhuang 050031, China; cppyjy@163.com; 2 Hebei Province Collaborative Innovation Center for Sustainable Utilization of Water Resources and Optimization of Industrial Structure, Shijiazhuang 050031, China; 3 Hebei Province Key Laboratory of Sustained Utilization and Development of Water Recourse, Shijiazhuang 050031, China; 4 Institute of Hydrogeology and Environmental Geology, Chinese Academy of Geological Sciences, Shijiazhuang 050061, China; ningzhuozhuo@163.com; 5 Key Laboratory of Groundwater Remediation of Hebei Province & China Geological Survey, Shijiazhuang 050061, China

**Keywords:** petroleum-contaminated aquifers, carbon sink effect, sulfur-oxidizing bacteria

## Abstract

Groundwater at petroleum-contaminated sites typically exhibits elevated dissolved inorganic carbon (DIC) levels due to hydrocarbon biodegradation; however, our prior field investigations revealed an enigmatic DIC depletion anomaly that starkly contradicts this global pattern and points to an unrecognized carbon sink. In a breakthrough demonstration, this study provides the first experimental confirmation that sulfur-oxidizing bacteria (SOB) drive substantial carbon sequestration via a coupled sulfur oxidation autotrophic assimilation process. Through integrated hydrochemical monitoring and 16S rRNA sequencing in an enrichment culture system, we captured the complete DIC transformation trajectory: heterotrophic acetate degradation initially increased DIC to 370 mg/L, but subsequent autotrophic assimilation by SOB dramatically reduced DIC to 270 mg/L, yielding a net consumption of 85 mg/L. The distinctive pH dynamics (initial alkalization followed by acidification) further corroborated microbial regulation of carbon cycling. Critically, *Pseudomonas stutzeri* and *P. alcaliphila* were identified as the dominant carbon-fixing agents. These findings definitively establish that chemolithoautotrophic SOB convert DIC into organic carbon through a “sulfur oxidation-carbon fixation” coupling mechanism, overturning the conventional paradigm of petroleum-contaminated sites as perpetual carbon sources. The study fundamentally redefines natural attenuation frameworks by introducing microbial carbon sink potential as an essential assessment metric for environmental sustainability.

## 1. Introduction

The biodegradation of petroleum hydrocarbon contaminants in groundwater has long been considered a carbon cycle process dominated by heterotrophic microorganisms. According to traditional theory, heterotrophic microorganisms utilize petroleum hydrocarbons as electron donors, converting organic carbon into dissolved inorganic carbon (DIC) through redox reactions, resulting in an increase in CO_2_ concentration in groundwater [1]. This mechanism has been verified through numerous case studies [2,3,4,5,6]. For instance, research by Suarez and Rifai on petroleum-contaminated sites along the Gulf Coast of the United States demonstrated a significant positive correlation between DIC concentration and the extent of petroleum hydrocarbon degradation [7]. Similarly, Maric et al. [8] observed abnormally elevated DIC levels at a refinery-contaminated site in Serbia. Such processes are often accompanied by the dissolution of carbonate rocks, further exacerbating DIC accumulation [9]. Consequently, elevated DIC concentrations are widely regarded as a critical indicator for assessing natural attenuation of petroleum contamination [10].

However, during the long-term monitoring of a petroleum-contaminated site in the North China Plain, our research team observed a phenomenon that contradicts conventional understanding: the DIC concentration in the core area of the contaminant plume was significantly lower than the background value, and this anomalous feature persisted over a two-year period [11]. This unexpected finding challenges traditional hydrogeochemical models, suggesting the existence of an unrecognized carbon sink mechanism. Through combined hydrochemical and microbial community analysis, the research team found that the abundance of chemolithoautotrophic bacteria such as *Hydrogenophaga* and *Pseudomonas* was higher in the contaminated area and negatively correlated with DIC concentration [12]. This led to the hypothesis that autotrophic microorganisms, through assimilation or induced precipitation, convert DIC into organic carbon or carbonate precipitates, which may be the key cause of the DIC anomaly [11,12]. Notably, a high abundance of sulfur-oxidizing bacteria (SOB), such as *Thiomargarita* and *Thiodictyon*, was found in the DIC anomaly area, implying their potential role in carbon fixation [12]. In addition, in recent years, an increasing number of studies have suggested that there are a substantial number of carbon sink microorganisms in soil contaminated by petroleum hydrocarbons, and that such soil is highly likely to be a carbon sink rather than a carbon source [13,14,15], but this has not been experimentally confirmed. Claudia Kellermann et al. identified sulfur-oxidizing bacteria with carbon fixation genes at petroleum-contaminated sites, but did not investigate their carbon fixation capabilities [16].

To confirm the presence of sulfur-oxidizing bacteria with carbon fixation capabilities in petroleum-contaminated aquifers and elucidate their role in DIC depletion anomalies, this study established controlled experiments using groundwater microorganisms from a typical contaminated site in the North China Plain. Specifically, we aimed to: (1) verify sulfur-oxidizing bacterial activity through sulfate generation dynamics; (2) characterize DIC transformation processes mediated by SOB via hydrochemical monitoring; (3) assess the carbon sink mechanism through microbial assimilation patterns. Through integrated hydrochemical monitoring, microbial community analysis, and enrichment experiments, this work provides empirical evidence for autotrophic SOB in regulating carbon cycling at petroleum-contaminated sites, refining natural attenuation assessment frameworks.

## 2. Materials and Methods

### 2.1. Experimental Materials

A SOB medium was prepared for the enrichment and cultivation of SOB. The specific formulation was as follows (g/L): NaH_2_PO_4_ 1.22, Na_2_HPO_4_ 1.39, NH_4_Cl 1, MgCl_2_ 0.1, FeCl_3_ 0.03, CaCl_2_ 0.03, MnCl_2_ 0.03, KNO_3_ 0.5, Na_2_S_2_O_3_ 6, NaHCO_3_ 2, CH_3_COONa 1. Additionally, 1 mL of a trace element mixture was added. After sterilization, the Na_2_S_2_O_3_ solution was filter-sterilized and added to the autoclaved medium (prepared without Na_2_S_2_O_3_). The pH of the medium was adjusted to approximately 7.0.

The composition of the trace element solution (g/L) was as follows: FeSO_4_·4H_2_O 1.80, CoCl_2_·6H_2_O 0.25, CuCl_2_·2H_2_O 0.01, NiCl·6H_2_O 0.01, MnCl_2_·4H_2_O 0.70, ZnCl_2_ 0.1, H_3_BO_3_ 0.5, (NH_4_)_6_Mo_7_O_24_·4H_2_O 0.10 [17].

The microorganisms for enrichment and cultivation were derived from water samples MW3 and MW4 collected from the petroleum-contaminated site in July 2018 [11,12,18].

### 2.2. Experimental Methods

First, sulfur-oxidizing bacteria were enriched and cultured. The specific steps were as follows: 95 mL of medium was dispensed into 150 mL conical flasks, sealed with breathable sealing film, and sterilized in an autoclave. After sterilization and cooling, 5 mL of filter-sterilized sodium thiosulfate solution and 1 mL of sample bacterial solution were added to each flask in a sterile environment. The flasks were sealed with breathable sealing film and placed in a 30 °C, 200 rpm constant temperature shaking incubator for dark oscillation culture. After 4 days of shaking, 1 mL of the culture liquid was taken and inoculated into a new flask containing 100 mL of freshly sterilized medium, continuing the culture as the second generation, and so on until the fifth generation.

The 5th-generation enriched culture was inoculated into fresh medium. Meanwhile, a control experiment was set up where no microbial inoculum was added, while all other conditions were kept the same. Each treatment and control were performed in triplicate. On days 0, 4, 8, 13, 18, and 25, the concentrations of DIC, pH, and SO_4_^2−^ were measured to analyze their trends. pH was determined using a pH meter (HACH-HQ11d, Loveland, CO, USA). Sulfate (SO_4_^2−^) concentrations were quantified via spectrophotometry (HACH-DR1900, Loveland, CO, USA). DIC was calculated from alkalinity measurements obtained through acid-base titration [19].

After the experiment, the microbial culture was filtered through a 0.22 μm pore-size membrane to collect microbial cells. DNA was extracted, and the 16S rRNA gene fragments were amplified using the 515F and 907R primers. Sequencing was performed on the Miseq platform at Majorbio Company (Shanghai, China). The microbial community structure was analyzed at the species level. Detailed sequencing and analysis methods can be found in the referenced literature [20].

## 3. Results

### 3.1. Dynamic Characteristics of Hydrochemical Indicators

**Figure 1:**
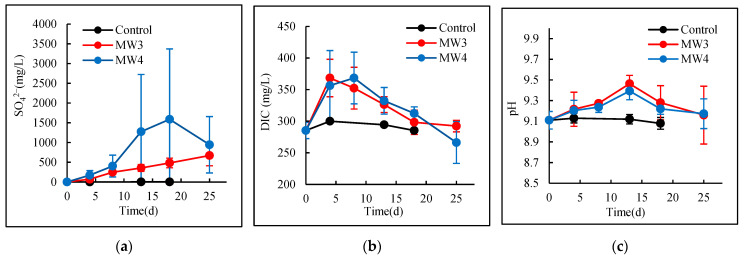
Variation curves of (**a**) SO_4_^2−^, (**b**) DIC, and (**c**) pH during sulfur-oxidizing bacterial metabolism.

The temporal dynamics of hydrogeochemical parameters (SO_4_^2−^, DIC, and pH) during the 25-day enrichment culture experiment are presented in Figure 1. Three distinct metabolic phases were identified through integrated analysis of microbial activity and geochemical shifts.

SO_4_^2−^ concentrations (Figure 1a) exhibited contrasting patterns between MW3 and MW4 cultures. In MW3, SO_4_^2−^ increased linearly from 0 to 670 mg/L (R^2^ = 0.987, *p* < 0.05) with minimal inter-replicate variation (CV < 40%), indicating stable sulfur oxidation activity. The constant oxidation rate suggests rapid bacterial population establishment (completed within 4 days) followed by sustained metabolic activity. Conversely, MW4 showed an initial SO_4_^2−^ increase (0–18 d: 215→480 mg/L) followed by a decline (18–25 d: 480→320 mg/L) with significant replicate variability (CV = 68–113%). This biphasic pattern likely resulted from transient oxygen depletion enabling sulfate-reducing bacteria activity.

DIC concentrations (Figure 1b) demonstrated a two-stage response in both cultures. The dynamics of MW3 and MW4 treatment groups exhibited statistically significant differences compared to the control group (*p* < 0.05). Although no significant inter-group variation (*p* > 0.05) was observed between MW3 and MW4, their evolutionary patterns demonstrated treatment-specific characteristics. During Phase I (0–5 d for MW3; 0–8 d for MW4), heterotrophic acetate degradation dominated, causing DIC to increase from 285 mg/L to ~370 mg/L (Δ + 85 mg/L). This process simulated petroleum hydrocarbon-derived DIC production in contaminated aquifers. Phase II showed marked DIC reduction: MW3 decreased to 300 mg/L (18 d) while MW4 declined to 270 mg/L (25 d), representing net DIC consumption of 55 mg/L and 115 mg/L, respectively. The final DIC concentration in MW4 dropped below initial levels (270 vs. 285 mg/L), indicating sustained autotrophic assimilation. A stabilization phase (MW3: 18–25 d) suggested equilibrium between DIC production (heterotrophic) and consumption (autotrophic) processes.

Regarding pH dynamics (Figure 1c), both MW3 and MW4 exhibited statistically significant differences compared to the control group throughout the experimental period (*p* < 0.05). However, no significant inter-group variation was detected between MW3 and MW4 treatments (*p* > 0.05). The pH changes in both MW3 and MW4 treatments exhibited a two-phase pattern: an increasing phase during days 0–13 followed by a decreasing phase after day 13.

### 3.2. Microbial Identification

**Figure 2:**
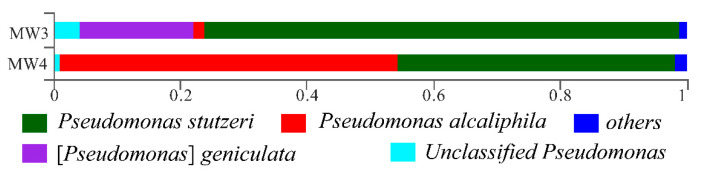
Population structure of sulfur-oxidizing bacteria of enrichment culture.

To identify the sulfur-oxidizing bacteria (group), their DNA was subjected to 16S rRNA gene sequencing analysis, and the population structure analysis results are shown in Figure 2.

As shown in Figure 2, the dominant bacterial species in MW4 are *Pseudomonas stutzeri* and *Pseudomonas alcaliphila* under the genus *Pseudomonas*, along with some unclassified *Pseudomonas* and other species. MW3 primarily contains *Pseudomonas stutzeri* and [*Pseudomonas*] *geniculate* under the genus *Stenotrophomonas*, with minor abundances of *Pseudomonas alcaliphila*, unclassified *Pseudomonas*, and other species. Both MW3 and MW4 share *Pseudomonas* as the dominant genus, with *Pseudomonas stutzeri* being highly abundant in both.

## 4. Discussions

### 4.1. Microbial and Biogeochemical Processes

This study successfully enriched a microbial consortium from petroleum-contaminated groundwater capable of utilizing reduced sulfur as an electron donor, which induced a significant decrease in DIC. These findings strongly suggest that such microorganisms are primary contributors to the observed low DIC anomalies in petroleum-contaminated aquifers. Sequencing analysis revealed that the enriched microbial consortium was predominantly composed of genus *Pseudomonas*, which is commonly detected in petroleum-contaminated environments and recognized for its petroleum hydrocarbon degradation capacity. Notably, certain species within this genus have been documented to exhibit sulfur oxidation capability [21] or carbon fixation capacity [22], demonstrating typical characteristics of facultative autotrophic microorganisms. At the species level, *Pseudomonas stutzeri* identified in this experimental system is renowned for its denitrifying degradation of organic compounds and sulfur oxidation capabilities, though no literature has explicitly confirmed its autotrophic metabolic functions [23,24,25]. According to available studies, *Pseudomonas alcaliphila*—the most dominant species in MW4—can also degrade various organic compounds, but its sulfur oxidation ability has not been reported [26]. The functional roles of these bacteria warrant further investigation. Previous analysis of groundwater microbial communities at the site revealed that *Pseudomonas* was not a dominant genus in contamination plume flanking monitoring wells MW3 and MW4, but exhibited predominance in the contamination source zone [20]. Although this genus may not prevail under generic petroleum-contaminated conditions, when subjected to selective cultivation in SOB enrichment media or alternative environments where adaptive capacity is demonstrated, their metabolic advantages in thiosulfate oxidation and DIC assimilation become amplified, enabling them to achieve ecological dominance. The contamination source zone likely provided favorable conditions for the proliferation of these microorganisms, thereby contributing to the observed low DIC anomalies. Concurrently, elevated pH values and reduced calcium/magnesium ion concentrations in the source zone compared to peripheral areas align with experimental observations. This correspondence supports the hypothesis that *Pseudomonas* likely executes carbon fixation at this site, further substantiating the proposition that autotrophic microbial activity drives DIC depletion [12].

However, it should be noted that the site may harbor additional carbon-fixing microorganisms beyond *Pseudomonas*, as our enrichment conditions were specifically optimized for this genus. Future studies should employ varied media formulations and experimental parameters to uncover a broader diversity of DIC-assimilating microorganisms.

Within this experimental system, DIC concentrations exhibited a biphasic trend of initial elevation followed by subsequent decline. This dynamic coincided with concurrent sulfur oxidation and reduction processes (notably sulfur reduction observed in MW4 at day 25). These observations suggest the simultaneous occurrence of microbial heterotrophic and autotrophic metabolism, which aligns with the documented facultative autotrophic capabilities of *Pseudomonas*.

In this cultivation system, heterotrophic metabolism primarily involves the mineralization of acetate, the fundamental process of which is as follows (Equation (1)) [24].

(1) $${\mathrm{CH}}_{3}{\mathrm{COO}}^{-}{+H}^{+}{+2O}_{2}\overset{\mathrm{Heterotrophic}\;\mathrm{metabolism}}{\to }{2\mathrm{CO}}_{2}{+2H}_{2}O⇌2{\mathrm{HCO}}_{3}^{-}{+2H}^{+}⇌2{\mathrm{CO}}_{3}^{2-}{+4H}^{+}$$

Under the culture conditions (pH 9.0–9.5), the majority of DIC exists in the form of HCO_3_^−^, with a small portion present as CO_3_^2−^ according to the carbonate balance [27,28]. Therefore, the H^+^ would be produced. Elevated pH conditions correlate with an increased proportion of carbonate ions, which is concomitant with enhanced acid production. Additionally, acetate degradation inherently produces acidic metabolites [29,30]. Concurrently, acetate hydrolysis originally contributes alkalinity to the solution. As acetate concentration decreases, the buffering capacity diminishes, leading to H^+^ accumulation. Considering the combined effects of the two aforementioned factors, the degradation of acetate ions (CH_3_COO^−^) results in a decrease in pH.

Autotrophic metabolism can be dissected into the oxidation of thiosulfate and DIC assimilation. The thiosulfate oxidation by microorganisms also contributes to acid production as follows (Equation (2)) [31,32,33,34].

(2) $$S_{2}O_{3}^{2-}{+2O}_{2}{+H}_{2}O\overset{\mathrm{Sulfur}-\mathrm{oxidizing}\;\mathrm{bacteria}}{\to }{2\mathrm{SO}}_{4}^{2-}{+2H}^{+}.$$

While the DIC assimilation process contributes to alkalinity production as follows (Equations (3) and (4)) [35,36,37].

(3) $$CO_{3}^{2-}+4H_{2}{O+4e}^{-}\overset{\mathrm{Autotrophic}\;\mathrm{metabolism}}{\to }CH_{2}O+6OH^{-},$$

(4) $$HCO_{3}^{-}+3H_{2}{O+4e}^{-}\overset{\mathrm{Autotrophic}\;\mathrm{metabolism}}{\to }CH_{2}O+5OH^{-}$$

Based on the analysis of the aforementioned processes, it is evident that only the DIC assimilation process can generate hydroxide ions (OH^−^), which directly leads to the increase in pH. In later stages, if the intensity of DIC assimilation weakens while other processes (such as acetate degradation and thiosulfate oxidation) become relatively dominant, the net result will be a decrease in pH.

Beyond microbial assimilation, methanogenesis and carbonate precipitation represent significant biogeochemical pathways for DIC reduction [38]. However, methanogenesis requires strictly anaerobic conditions, which are incompatible with the experimental set up of this study.

Carbonate precipitation serves as a crucial carbon sink mechanism, requiring two prerequisites: (1) the presence of divalent metal cations (e.g., Ca^2+^, Mg^2+^) as hardness ions, and (2) DIC concentration or environmental pH reaching supersaturation levels. While the cultivation medium used in this study contained trace amounts of Ca^2+^, Mg^2+^, and Fe^2+^ ions—providing a material basis for precipitation—their limited concentrations typically only permit minor precipitation when DIC levels or pH values rise significantly. It is widely recognized that alkaline microenvironments or macroenvironments created by microbial metabolic activities (particularly autotrophic microorganisms) are pivotal for inducing carbonate precipitation. Consequently, this process is often categorized as autotrophic and microbe mediated.

In summary, within this experimental system, the decline in DIC concentration is predominantly driven by autotrophic carbon fixation through assimilation and microbially induced carbonate precipitation. Notably, even when precipitation occurs, its initial driving force remains attributable to autotrophic microbial carbon fixation.

In petroleum-contaminated sites, petroleum hydrocarbons as organic matter do not hydrolyze to produce alkalinity. Meanwhile, the absence of acid-base buffering components in groundwater, such as sodium dihydrogen phosphate (NaH_2_PO_4_), disodium hydrogen phosphate (Na_2_HPO_4_), ammonium chloride (NH_4_Cl), and sodium acetate (CH_3_COONa), exacerbates the intensity of pH fluctuations. However, the presence of hardness ions like calcium (Ca^2+^) and magnesium (Mg^2+^) in groundwater implies that elevated pH will promote the formation of carbonate precipitates (e.g., CaCO_3_, MgCO_3_) through reactions with DIC, which constitutes another significant carbon sink pathway.

### 4.2. Limitations Analysis and Future Prospects

This study successfully revealed the mechanism of DIC dynamics mediated by SOB using an enrichment culture system. However, it is important to recognize that this experimental system differs significantly from actual aquifer conditions: the artificially elevated substrate concentrations substantially amplified microbial activity, far exceeding typical metabolic levels observed in contaminated groundwater environments. The phosphate buffering system constrained pH, suppressing its natural fluctuations; in contrast, actual aquifers lack such buffers and can exhibit considerable pH variations that may drive different biogeochemical processes. Furthermore, the use of aerobic shaking cultivation replaced the natural anaerobic environment of the site, potentially leading to shifts in electron acceptor preferences and alterations in the native microbial community structure, thus not fully reflecting the in situ state.

Nonetheless, given the complexity of directly replicating the phenomenon of DIC depletion in petroleum-contaminated sites and the failure of our earlier attempts (primarily due to insufficient understanding of the in situ environmental and nutritional conditions), the approach employed in this study remains a preferred method for elucidating the core mechanism of this phenomenon. Consequently, we designed a simplified yet effective pathway: utilizing microorganisms sourced from the contaminated site as the primary actors, employing acetate—an easily utilizable intermediate of petroleum degradation—as a proxy for the complex organic components present in situ, and focusing on the dominant SOB identified through metagenomic analysis as the target process. Through this experimental design, conducted in SOB enrichment media under aerobic conditions, we successfully accelerated the recreation of the core field observation—significant DIC depletion. This research not only directly confirmed that the site’s SOB, predicted via metagenomics, possesses genuine carbon fixation capability under enrichment, preliminarily clarified the role of SOB as a carbon sink mechanism in petroleum-contaminated environments, but also opened a crucial path for a deeper understanding of carbon cycling in contaminated groundwater systems.

This study significantly enhanced the pool of information concerning carbon-fixing microorganisms, particularly SOB, in petroleum-contaminated sites and deepened the understanding of the specific environmental and nutritional conditions required by these microorganisms for carbon fixation. It provides an essential foundation and new perspectives for subsequent research on hydrocarbon-utilizing carbon-fixing microorganisms. Building upon the limitations and key findings of the current research, future work will focus on conducting microcosm simulation experiments. These experiments aim to create systems more closely resembling actual conditions in petroleum-contaminated sites: using actual site groundwater (without strong buffers) under anaerobic conditions (utilizing nitrate, Fe(III) as electron acceptors), while specifically investigating the influence of electron donors like hydrogen and the role of petroleum hydrocarbons themselves as carbon sources. This approach is designed to uncover microbial carbon fixation mechanisms occurring under more realistic conditions characterized by low-molecular-weight organic carbon sources, anaerobic environments, and dynamic pH. Ultimately, this enhanced understanding of processes relevant to actual field conditions will provide a solid theoretical foundation for the scientific and rational assessment of natural attenuation in petroleum contamination and for the accurate accounting of carbon source-sink balances at contaminated sites.

### 4.3. Practical Implications

The confirmed carbon sink mechanism mediated by SOB presents transformative potential for environmental management applications.

In the context of monitoring: historically, elevated DIC concentrations often signal organic contamination. However, this study reveals that relying solely on DIC as an indicator of contamination or as a metric for degradation assessment requires caution. SOB-mediated carbon fixation can significantly deplete DIC, potentially leading to misinterpretations underestimation of ongoing degradation or even missing pollution signals entirely where fixation is active. Consequently, integrating these microbial carbon sink processes could significantly refine existing natural attenuation assessment frameworks.

Regarding remediation technologies, the study suggests a novel pathway: supplementing environments with key electron donors essential for SOB-driven carbon fixation, such as thiosulfate or hydrogen. This strategy could stimulate both contaminant degradation and enhance carbon sequestration via assimilation and precipitation, providing a proof-of-concept for synergistic pollution reduction and carbon capture approaches. While the transformation of these concepts into practical monitoring and remediation tools demands further development and validation through deeper theoretical investigations and field trials, this work serves as a crucial initial exploration highlighting their feasibility and the tangible value of understanding microbial carbon sinks in contaminated environments.

## 5. Conclusions

Emerging evidence reveals previously overlooked carbon sequestration potential within petroleum-contaminated aquifers mediated by chemolithoautotrophic microbial communities. Notably, SOB exhibits remarkable capacity for DIC assimilation through autotrophic metabolism while generating alkaline byproducts. This microbially induced alkalinization facilitates carbonate mineral precipitation, synergistically reducing aqueous DIC concentrations and potentially creating characteristic DIC depletion anomalies. Although the current experimental model employed enriched SOB consortia under controlled aerobic conditions, diverging from in situ microbial community composition and anaerobic groundwater environments, it fundamentally deciphers the biogeochemical coupling driving carbon fixation processes. Crucially, this work establishes a novel paradigm where microbial cross-feeding between heterotrophic hydrocarbon degraders and autotrophic SOB creates net carbon sink conditions, challenging the conventional perception of hydrocarbon-impacted aquifers as perpetual carbon sources. However, this study represents only preliminary research in the investigation of hydrocarbon degradation and carbon fixation interactions at petroleum-contaminated sites. Several critical knowledge gaps remain to be addressed, particularly regarding the following: (1) the complex microbial community dynamics governing these coupled processes, (2) the environmental factors controlling their relative contributions, and (3) the long-term stability of carbon sequestration under field conditions. Future research should prioritize multi-parameter microcosm experiments that more closely simulate actual site conditions (e.g., anaerobic environments with native electron acceptors, dynamic redox fluctuations, and representative hydrocarbon mixtures). Such systematic investigations will be essential for developing a comprehensive theoretical framework that accurately describes natural attenuation processes while incorporating microbial carbon sink mechanisms into modern carbon cycling models.

## Data Availability

The data presented in this study are available on request from the corresponding author, data are not publicly available due to privacy.
